# Clinical and genetic analysis of recurrent adult-type granulosa cell tumor of the ovary: Persistent preservation of heterozygous *c*.*402C>G FOXL2* mutation

**DOI:** 10.1371/journal.pone.0178989

**Published:** 2017-06-08

**Authors:** Satoshi Yanagida, Michael S. Anglesio, Tayyebeh M. Nazeran, Amy Lum, Momoko Inoue, Yasushi Iida, Hirokuni Takano, Takashi Nikaido, Aikou Okamoto, David G. Huntsman

**Affiliations:** 1 Department of Obstetrics and Gynecology, University of British Columbia, Vancouver, British Columbia, Canada; 2 Department of Pathology and Laboratory Medicine, University of British Columbia, Vancouver, British Columbia, Canada; 3 Department of Obstetrics and Gynecology, The Jikei University School of Medicine, Tokyo, Japan; 4 Department of Molecular Oncology, BC Cancer Agency Research Centre, Vancouver, British Columbia, Canada; 5 Department of Pathology, Kosei General Hospital, Tokyo, Japan; Indiana University School of Medicine, UNITED STATES

## Abstract

**Background:**

Adult-type granulosa cell tumors of the ovary (aGCTs) are rare tumors that represent 2–5% of ovarian malignancies. The prognosis of this tumor is favorable, and it is characterized by slow progression. 10–30% of these tumors recur after 4–7 years of the primary surgery and the 5-year survival rate from the first recurrence is 55%, for the incompletely resected patients. At this time, complete resection is the only prognostic factor for better outcome, and establishing a novel strategy for identification and/or treatment of recurrent tumors is crucial. After the discovery of heterozygous *c*.*402C>G FOXL2* mutations in 97% of cases of aGCT, much effort has been made to find the role of the mutation on the pathogenesis of aGCT, however, little is known about the role of the mutation in disease progression.

**Methods:**

We analyzed the clinical data of 56 aGCT patients to find a marker of recurrence. In particular, we compared the *FOXL2* status in 5 matched primary and recurrent samples by immunohistochemistry, and TaqMan allelic discrimination assay to address the role of *FOXL2* in potential mechanisms of recurrence.

**Results:**

The clinical data analysis was consistent with complete resection as an indicator of disease eradication, though the sample size was limited. The genetic analysis showed all the samples, including recurrent tumor samples up to 14 years after the primary surgery, expressed heterozygous *c*.*402C>G FOXL2* mutation and the FOXL2 protein expression.

**Conclusion:**

This report describes the preservation of heterozygous *c*.*402C>G FOXL2* mutation in recurrent aGCTs. This finding adds further credence to the concept that the *c*.*402C>G FOXL2* mutation is oncogenic and integral to this disease.

## Introduction

Granulosa Cell Tumor of the ovary (GCTs) is the most clinically significant type of sex-cord stromal tumor of the ovary and accounts for 2–5% of overall ovarian malignancies[1–3]. GCT are divided into two distinct histologic subtypes, adult type (aGCTs) (95%), and juvenile type (5%) by histologic features. aGCTs are defined in most cases by the presence of a specific *c*.*402C>G* missense mutation in the forkhead transcription factor *FOXL2* [4][5].

This tumor is characterized by its relatively indolent behavior, compared to epithelial ovarian cancers. Prior to the finding of the *c*.*402C>G* mutation in *FOXL2* and use of a molecular definition of this disease[4][5], literature reports suggested a recurrence rate of 10–30% and a median time to the first recurrence of 4–7 years [1][6][7], whereas most of the epithelial ovarian cancers recur within two years after primary surgery. A major issue in research on aGCTs is the difficulty of accumulating the number of samples because of its rarity and indolent behavior. Because of these characteristics, this tumor requires especially long term follow up [6][8].

Despite the challenge of collecting sufficient patient cohorts, many researchers have attempted to define prognostic factors of this tumor and suggest treatments to prevent the recurrence. In studies with larger cohorts[1][6][7][9][10], 80–90% of the tumors were diagnosed in early stage and the 10-year survival rates were 94.8%[7]. The initial stage, complete resection of tumor, mitotic rate, and nuclear atypia were the predominant prognostic factors[6][7][9][10]. The efficiency of the chemotherapy is controversial, but platinum-based chemotherapy such as BEC-regimen (bleomycin, etoposide, and carboplatin) or paclitaxel-carboplatin have been used for incompletely resected advanced patients [1][6][10][11]. A recent analysis of clinicopathological markers from Farkkila and colleagues showed that high expression of GATA4 and HER2 were prognostic of shorter disease-free survival in low-stage aGCTs [12]. Colin and colleagues also showed reduced β-catenin expression in primary tumors correlated with increased risk of recurrence [13].

Despite an overall favorable 10-years survival rate for aGCT, the prognosis after relapse is still poor. In previous studies, 30% (3/10) [1] and 25.7% (9/35) [9] of recurrent patient died of disease, and the 5-year overall survival rate from the first recurrence for patients, with or without residual tumor at the secondary debulking surgery, was 55.6% and 87.4% [9]. Examination of recurrent patients after debulking surgery and platinum-based chemotherapy suggests only the absence of the residual tumor is prognostic and the effectiveness of the platinum-based chemotherapy is unclear [6][7][9]. Anecdotal case reports suggest some efficacy for aromatase inhibitors and Cytochrome P17 Inhibitor [14–16], while Xia and colleagues showed Bevacizumab therapy yielded the response rate of 38% and a clinical benefit of 63% [17]. Clarifying and validating a novel strategy for treatment of recurrent tumors is critical.

In 2009 Shah et al. described the somatic *c*.*402C>G* missense mutation in the *FOXL2* gene in 97% of aGCTs tumors, this strongly implied a driver potential for this mutation in aGCT[4]. Since then, many researchers have attempted to describe the molecular functions or target genes of this mutant transcription factor and its effect on tumorigenesis [18–26]. However, the impact of this mutation on mechanisms of recurrence amongst aGCTs has not been addressed.

The aim of this study is to analyze prognostic clinicopathological variables and investigate the *FOXL2* status in primary and recurrent tumors to better understand the function of this gene in recurrence. This is the first report describing the *c*.*402C>G FOXL2* mutation is maintained even in exceptionally late-recurrence tumors.

## Material and methods

### Patient and samples

This study protocol was approved by the Ethics Committee for Biomedical Research of the Jikei Institutional Review Board, Jikei University School of Medicine, Tokyo, Japan. Each patient provided written informed consent for this research. All investigations were performed in accordance with a protocol approved by the ethic committee at BC Cancer Agency, Vancouver General Hospital, Vancouver, Canada.

The clinical data of 56 patients who underwent surgical treatment for aGCTs at Jikei University and related hospital in Japan between 1990 to 2014 were included in this analysis. Tumors were staged in accordance with the International Federation of Gynecology and Obstetrics (FIGO) system (1988).

For patients with recurrent tumors, we obtained available formalin-fixed paraffin-embedded(FFPE) tumor tissues from cytoreductive surgeries for both primary and recurrent tumors at the Department of Pathology, Jikei University Hospital (19 samples from 5 patients: one or more specimens from the initial/primary tumor, plus metastatic sites if available, and at least one recurrent tumor specimen per patient). Progression free survival (PFS) was calculated as months from the date of previous cytoreductive surgery to the following surgery.

### Pathological review

The hematoxylin and eosin-stained sections of all recurrent patients were independently reviewed by two specialist gynecologic pathologists (TMN, TN) before mutational analysis. Pathologists were blinded to genomic data.

### Immunohistochemistry (IHC)

FOXL2 IHC was done using primary goat polyclonal FOXL2-antiserum (1:200; Imgenex, San Diego, CA, USA) and a secondary antibody (unconjugated rabbit anti-goat, Jackson ImmunoResearch Labs, West Grove, PA, USA) at 1:300. Scoring of FOXL2 IHC was performed by specialist gynecological pathologist (TMN) as described previously [4][27]. Tumors were divided into 4 groups; no staining (no positive cells: score 0), weak staining (0–30% of positive cells: score 1), moderate staining (30%-80% positive cells: score 2), and strong staining (80%< positive cells: score 3). IHC for p53 was performed using standard methods described previously and where abnormal staining patterns (absent/0 and strong/2) correlate near perfectly with mutation status[28][29][30].

### DNA isolation

Tumor areas were enriched by manual macro-dissection with a scalpel from 30μm thick sliced FFPE sections to remove non-tumor tissues; a pathologist-marked serial H&E-stained section was used as a guide. Samples were deparaffinized and genomic DNA was purified using QIAamp DNA FFPE Tissue Kit (Qiagen) following the manufacturer’s protocol.

### TaqMan allelic discrimination assay

TaqMan real-time PCR based allelic discrimination assay was performed according to procedural guidelines outlined previously [27] to genotype the *FOXL2 c*.*402C>G* mutation using primers: 5’-GCGCAAGGGCAACTACTG-3’ (forward) and 5’-CGGTAGTTGCCCTTCTCGAA-3’ (reverse), along with wild type specific probe (5’-FAM dye- CATGTCTTCCCAGGCCG-NFQ (non-fluorescent quencher)) and mutation specific probe (5’-VIC dye-CATGTCTTCGCAGGCCG-NFQ) included in the genotyping master mix. Reactions were performed in a 7900HT Fast Real-Time PCR System (Applied Biosystems). Reaction volume of 5 *μ*l was used for each replicate well including 2.5 *μ*l 2X TaqMan master mix (Life Technologies), 0.125 *μ*l (40 ×) custom synthesized allelic discrimination primer/probe mix (Life Technologies), 1 *μ*l DNA (standard input of 20 ng was adjusted), and water. After denaturation at 95°C for 10minutes, DNA was amplified over 40 cycles (95°C 15seconds, 60°C 1minute) [4][27]. DNA from molecularly diagnosed aGCT sample in the preceding study[4] was used as a positive control and the distilled water as a negative control. Each sample were duplicated.

### Statistical analysis

The statistical analysis was performed using Stat Mate V software (ATMS, Tokyo, Japan). P<0.05 was defined as statistically significant; all tests were 2-tailed. The survival curves were evaluated by the Kaplan-Meier method and the resulting curves were compared using the log-rank test. The multivariate analysis was performed using Cox’s regression model. Wilcoxon rank sum test was used for comparing IHC scores.

## Results

### Patient characteristics and the predictive marker for recurrence

The clinical information of all the patients are summarized in S1 Table. The median age of all patients was 46.5 years old (range 30–88 years old) (Table 1). Fifty-two patients (92.8%) were FIGO stage I, 1 patient (1.8%) stage II, and 3 patients (5.3%) stage III at diagnosis. All patients had received initial surgery: 33 patients (58.9%) had total abdominal hysterectomy and 8 patients (14.2%) had lymphadnectomy, one (1.8%) of them presented with residual tumor after surgery. All the 4 stage II or III patients (7.1%) had undergone platinum-based chemotherapy whereas none of stage I patient had. The median follow-up period was 86.9 months (range 1.2–285 months). No deaths were recorded among these patients and only 7 were lost to follow up.

**Table 1:** Patient characteristics.

Characteristics (n = 56)			N	%
Age	Median		46.5 (range 30–88)	
	≦50		34	60.7
	>50		22	39.2
Tumor size (cm)	Mean		10.8 (range 2–30)	
	<10		28	50
	≧10		28	50
Stage	I (Ia/Ic)/II		52 (44/8)/1	94.6
	III (IIIb/IIIc)		3 (1/2)	5.3
Residual tumor		Yes	1	1.8
		No	55	98.2
Type of surgery	Hysterectomy	Yes	33	58.9
		No	23	41
	Lymphadenectomy	Yes	8	14.2
		No	48	85.7
Chemotherapy		Yes	4*	7.1
		No	52	92.8
Median follow up period (months)	86.9 (1.2–285)			

*: DC (Docetaxel and Carboplatin) x1 case, EP (Etoposide and Cisplatin) x 2cases, BEP (Bleomycin, Etoposide and Cisplatin) x1 case

There were 7 recurrent patients (12.5%) during follow up (Table 2). The median age of recurrent patients was 50 years (range 32–66 years old). Five patients were FIGO stage I (5/52, 9.6%), and two were stage III (2/3, 66.7%) at initial surgery. One of the stage III patients had residual tumor. The median time to relapse was 112 months (range 31–172 months). Pelvis was the most common site of recurrence.

**Table 2:** Clinical findings of patients with recurrent disease.

No.	Age	Stage	Residual tumor	Initial surgery	Adjuvant chemotherapy	PFS (months)	Recurrent site	Treatment for recurrence	status	Follow up period (months)
#1	32	Ia	―	Oophorectomy	―	83	Abdominal and Pelvis	TAH+BSO+OMTX+ Tumorectomy	NED	166
#2	36	IIIc	<1cm	TAH+BSO+OMTx+ Tumorectomy	DC	32	Pelvis	Tumorectomy	AWD	121
#3	59	IIIb	―	TAH+BSO+OMTX+ Para Aorta-Pelvic LNX	EP	172	Abdominal	Tumorectomy	AWD	228
#4	50	Ic(a)	―	LSO	―	112	Pelvis	TAH+RSO+ OMTX+HAR	NED	154
#5	66	Ia	―	TAH+BSO+OMTX	―	31	Abdominal and Pelvis	Tumorectomy	AWD	81
#6	52	Ia	―	TAH+BSO	―	168	Pelvic LN	Tumorectomy	AWD	285
#7	42	Ia	―	LSO	―	147	Pelvis	SRH+RSO+OMTX+LAR+ Diaphragm peritonectomy	NED	154

TAH: total abdominal hysterectomy, BSO: bilateral salpingo-oophorectomy, OMTX: omentectomy, LNX: lymphadenectomy, LSO: left salpingo-oophorectomy, LAR: low anteriol resection, HAR: high anterior resection, DC: docetaxel+carboplatin, EP: VP16+CDDP, AWD: alive with disease, NED: no evidence disease

Age, stage, residual tumor, type of surgery, adjuvant chemotherapy, and tumor size were examined as prognostic markers of recurrence. Kaplan-Meier curve (S1 Fig) and Univariate (Table 3) analysis revealed the residual tumor at the primary surgery was predictive of recurrence. Consistent with previous reports [6][8], multivariate analysis also showed residual tumor was the only predictive marker for the recurrence (Table 4).

**Table 3:** Risk factors for recurrence (Univariate analysis).

Characteristics			n (%)	P-value
Age	≦50		34 (60.7)	0.29
	>50		22 (39.2)	
Tumor size (cm)	<10		28 (50.0)	0.25
	≧10		28 (50.0)	
stage	I, II		53 (94.6)	0.12
	III, IV		3 (5.3)	
Residual tumor		No	55 (98.2)	0.00001
		Yes	1 (1.8)	
Type of surgery	Fertility-sparing	No	33 (58.9)	0.65
		Yes	23 (41.0)	
chemotherapy		No	52 (92.8)	0.17
		Yes	4 (7.1)	

**Table 4:** Risk factors for recurrence (multivariate analysis).

Characteristics			n (%)	HR (95%CI)	P-value
Age	≦50		34 (60.7)	6.47 (0.55–75.9)	0.13
	>50		22 (39.2)		
Tumor size (cm)	<10		28 (50.0)	1.51 (0.21–10.5)	0.69
	≧10		28 (50.0)		
stage	I, II		53 (94.6)	0.58 (0.041–8.33)	0.69
	III, IV		3 (5.3)		
Residual tumor		No	55 (98.2)	234.5 (2.15–25520)	0.023
		Yes	1 (1.8)		
Type of surgery	Fertility-sparing	No	33 (58.9)	2.84 (0.28–28.4)	0.67
		Yes	23 (41.0)		

### Protein expression and genotyping of *FOXL2*

#### Immunohistochemistry (IHC) on recurrent GCTs

**Fig 1:**
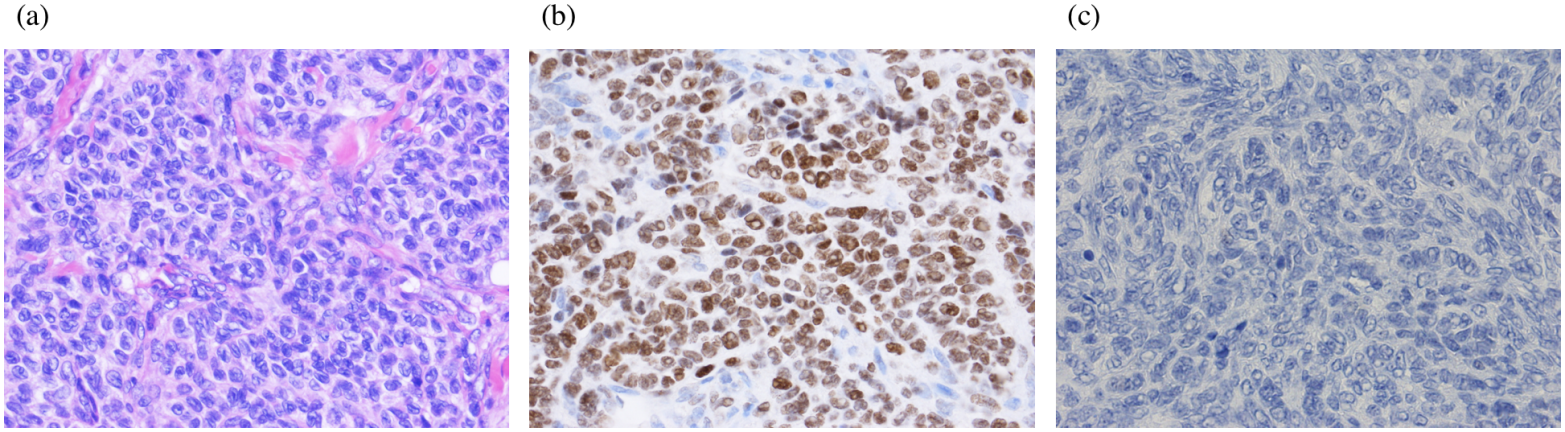
The expression of *FOXL2 and p53*. (a) H&E: The histological findings of aGCTs including small bland cells with the central round nuclei. The typical coffee bean nuclei with nuclear groove is seen. (b) FOXL2 was expressed in the nuclei of GCTs that were heterozygous c.402 C>G mutation. (c) No p53 staining was found in the sample of Pt #1-#4.

**Fig 2:**
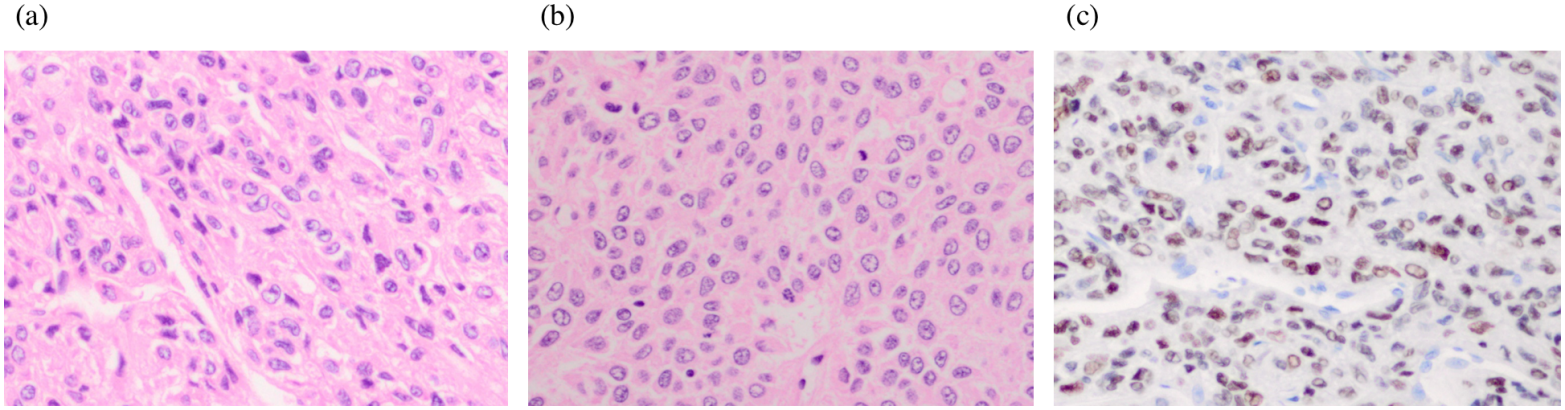
Histological finding and p53 expression of Pt #5. (a) The primary tumor of the Pt #5 revealed the bland tumor cells with variably nuclear grooves and a predominant diffuse pattern with minor trabecular and insular pattern which compatible with aGCTs. The mitotic activity was inconspicuous. (b) The recurrent tumor of the Pt #5 showed the similar histological findings as the primary tumor, though the mitotic activity was slightly higher compared to the primary tumor. (c) Primary and recurrent samples from patient #5 showed the moderate to strong p53 staining in the nucleus.

All the tumors were diagnosed as aGCTs (Figs 1a, 2a and 2b). IHC of recurrent tumors revealed all samples to be moderately to strongly positive for FOXL2 expression (Fig 1b). The samples from patient #1–4 showed normal p53 staining (Fig 1c), but all samples from patient #5 showed strong/abnormal tp53 nuclear accumulation (Fig 2c). This strong/abnormal p53 staining has been shown to be a highly accurate predictor of TP53 mutation in ovarian carcinomas [29][30]. The mean FOXL2 IHC score revealed no significant difference between primary and recurrent tumors, and between p53 normal and abnormal/strong staining tumors (Table 5, S2 Fig).

**Table 5:** FOXL2 status in recurrent aGCTs.

Patient No	age	stage	primary or recurrent	PFS (months)	sample No	site	H&E	FOXL2 IHC score	p53 IHC score	Heterozygous 402C>G mutation
1	32	Ic	primary	-	1	left ovary	aGCT	3	0	+
			1^st^ rec	83	2	right ovary	aGCT	3	0	+
					3	omentum	aGCT	3	0	+
					4	S-colon	aGCT	2	0	+
			2^nd^ rec	33	5	abdomen	aGCT	3	0	+
			3^rd^ rec	21	6	abdomen	aGCT	3	0	+
2	36	IIIc	primary	-	7	left ovary	aGCT	3	0	+
					8	omentum	aGCT	3	0	+
					9	para-bladder	aGCT	3	0	+
			rec	32	10	abdomen	aGCT	3	0	+
3	59	IIIb	primary	-	11	left ovary	aGCT	3	0	+
					12	right ovary	aGCT	3	0	+
			rec	172	13	colon	aGCT	3	0	+
4	50	Ic(a)	primary	-	14	left ovary	aGCT	2	0	+
					15	left ovary	aGCT	3	0	+
			rec	112	16	para-rectum	aGCT	3	0	+
5	66	Ia	primary	-	17	left ovary	aGCT	3	3	+
					18	left ovary	aGCT	2	2	+
			rec	31	19	abdomen	aGCT	3	3	+

PFS: Months from the date of previous cytoreductive surgery to the following surgery.

#### Allelic discrimination assay

**Fig 3:**
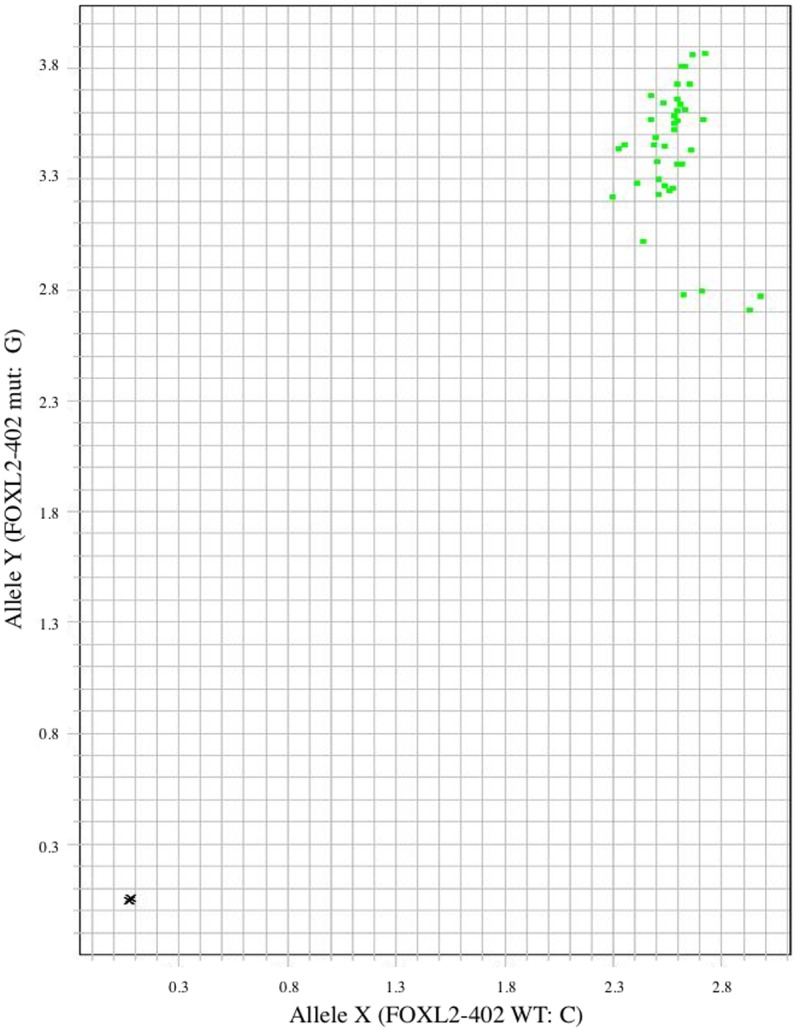
TaqMan based allelic discrimination assay. All samples gave fluorescent signal from both WT and mutant probes consistent with heterozygous c.402 C>G FOXL2 mutation. Each sample was duplicated and the black dot represents the negative control (H2O).

All tumors showed similar ratio of signal from both wild type (WT) and mutational (mut) allele in keeping with samples being heterozygous for the *c*.*402C>G FOXL2* mutation (Fig 3, S2 Table).

#### *FOXL2* status in recurrent aGCTs

The clinical characteristics and the *FOXL2* status in each recurrent patient and each sample are summarized in Table 5.

## Discussion

The rarity and slow progression of aGCTs has made it difficult to analyze large number of the patients and run clinical trials. Whilst initial stage, complete resection of tumor, mitotic rate, and nuclear atypia are the predominant prognostic factors for aGCT, no effective treatments, especially for recurrent tumors and aside from complete resection, have been established. For premenopausal women, fertility sparing surgery, defined as the preservation of the uterus and one ovary, is an option [1][7]. In a recent publication, we demonstrated among patients with a primary histologic diagnosis of aGCT almost 75% of deaths within the first five years occurred in patients who were misdiagnosed and never had this cancer[5]. Nevertheless, as the 5-years and 10-year survivals of molecularly defined aGCTs after relapse are 76.4% and 53.6% respectively, better management strategies for recurrent aGCTs are still needed [5] and research is needed to better understand such tumors.

Though intratumoral heterogeneity is seen in epithelial ovarian cancers[31–36], relatively little is known about the recurrent aGCT[37]. It is possible that less heterogeneity will be seen in aGCTs as they are cytogenetically simple and clinicopathologically homogenous [4] and not all aGCT patient undergo post-surgical platinum-based chemotherapy which is potentially mutagenic [38]. A recent publication by Zannoni and colleagues showed the *c*.*402C>G FOXL2* mutation was found in 89% (33/37) of primary and 80% (4/5) of metastatic aGCTs [37], albeit perfect concordance with mutant status between primary and metastasis (i.e. the 4 cases with *c*.*402C>G* in the primary also showed mutation in the metastatic lesion). However, to date no report has examined persistence or heterogeneity of FOXL2 status in recurrent aGCTs. Understanding heterogeneity in the context of recurrent aGCTs is important, herein consistent preservation of the heterozygous mutation in primary and recurrent lesions supports a view that *c*.*402C>G FOXL2* is indeed a driver gene for aGCT and this tumor retains dependence on the functions of the mutant protein.

The finding of pathognomonic *c*.*402C>G FOXL2* driver mutations in aGCTs has changed the way this cancer is viewed and opened opportunities to better understand its pathogenesis [18–26]. Herein we have shown the persistence of this mutation across the course of disease which suggests that this mutant protein or the as yet unknown pathways it abrogates could be useful targets for the management of metastatic aGCT.

In our study, the analysis of 56 aGCT patients (maximum following up time 24 years) revealed the recurrence rate was 12.5% (7/56) and the mean and median time to the first recurrence were 106 and 112 months. No deaths were found amongst recurrent patients.

The univariate and multivariate analysis supports the residual tumor after primary surgery as the only predictive marker for recurrence. These results are concordant with previous studies [5,6], however in our cohort this residual disease is apparent only in a single patient and confirmation on larger sample cohorts are needed. Overall, the importance of complete resection at the primary surgery cannot be understated.

### *c*.*402C>G FOXL2* mutation is critical for the tumorigenesis of aGCTs

To elucidate the role of *FOXL2* in the mechanism of recurrence, we stepped further and investigated the *FOXL2* status by comparing 19 samples from 5 patients, including multiple recurrences and metastatic lesions (Table 5).

All the primary tumor samples showed moderate to strong expression of FOXL2 in IHC, and heterozygous *c*.*402C>G FOXL2* mutation in the allelic discrimination assay. These finding is in agreement with the recent publication by McConechy and colleagues showing the diagnosis of aGCTs can be challenging and the frequency of *FOXL2* mutation in aGCT series should be over 90% [5]. With respect to p53: primary and recurrent samples from only patient #5 showed abnormal/strong expression, a pattern associated with TP53 mutation[29][30]. Patient #5 was diagnosed as aGCT and had somatic *c*.*402C>G FOXL2* mutation, but was clinically unique due to an exceptionally short time to recurrence (3 months), despite being apparently low stage Ia. Whether this unusually aggressive course was related to the p53 abnormalities is unknown.

All the recurrent samples also showed similar FOXL2 level of staining and heterozygous *c*.*402C>G FOXL2* mutation, between primary and recurrence, even in with late recurrence 14 years after primary surgery. One patient had three distinct recurrence events, with consistent results (FOXL2 IHC and mutation) in all specimens. No previous reports have focused on the *FOXL2* status in pairs of primary-recurrent tumors, however our result is concordant with the finding that 3 of 4 index cases and 16 of 69 in first series in original description of *c*.*402C>G FOXL2* by Shah and colleagues[4] were in fact recurrent tumors, and all of which expressed this mutation. Two of those series were matched samples though the time to the recurrence was not presented. In the present study, patient #2 and #3 had metastatic tumors in the primary surgery. These patients expressed heterozygous c.402C>G FOXL2 mutation both in the primary and metastatic lesions, matching findings of Zannoni and colleagues [37]. The finding that heterozygous *c*.*402C>G FOXL2* mutation and the FOXL2 protein expression were preserved in all evaluated primary, metastatic lesions, and recurrent samples is consistent with this mutation being critical for long-term maintenance of aGCTs, as opposed to strictly tumor initiation, and may also reflect the genetically stable characteristics of aGCTs.

## Conclusions

This is the first report to identify heterozygous *c*.*402C>G FOXL2* mutation as an apparent driver of aGCT in primary and matched recurrent tumors. The importance of the complete resection is evident in our cohort, despite its limited sample size. Larger cohorts, long term follow up and molecular analysis will be required to better this disease.

## Supporting information

S1 Table Summary of the clinical background of all patients. (TIFF)

S2 Table Summary of Allelic discrimination assay. (TIFF)

S1 Fig Kaplan-Meier curve of the patients with and without residual tumor. (TIFF)

S2 Fig FOXL2 IHC scoring. (TIFF)
