# Supplementary figures and images for: Clinical and genetic analysis of recurrent adult-type granulosa cell tumor of the ovary: Persistent preservation of heterozygous c.402C>G FOXL2 mutation
Source: PLoS One. 2017 Jun 8;12(6):e0178989. doi: 10.1371/journal.pone.0178989 (PMC5464638; doi:10.1371/journal.pone.0178989)

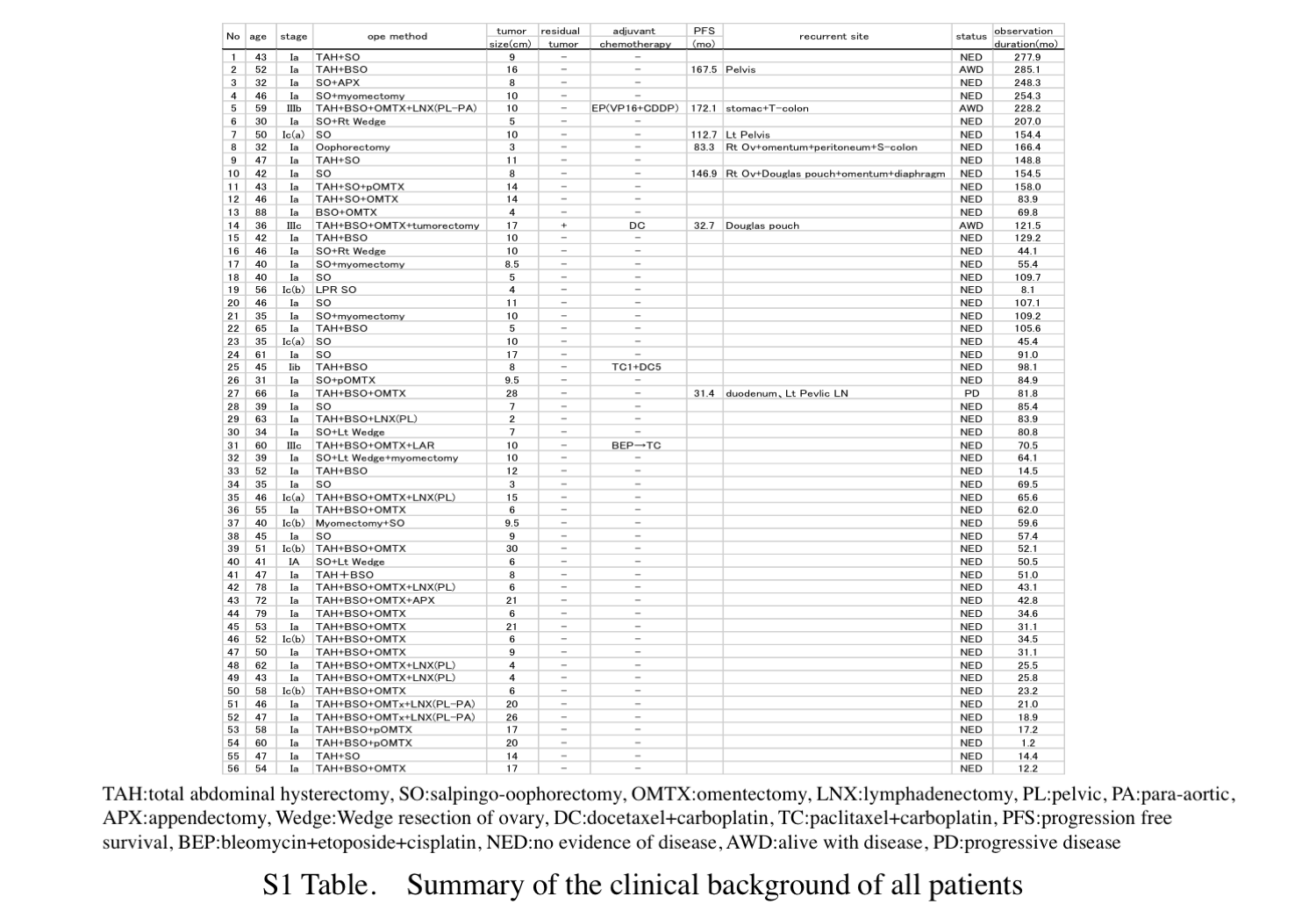

Supplement: S1 Table — (TIFF) [file pone.0178989.s001.tiff]

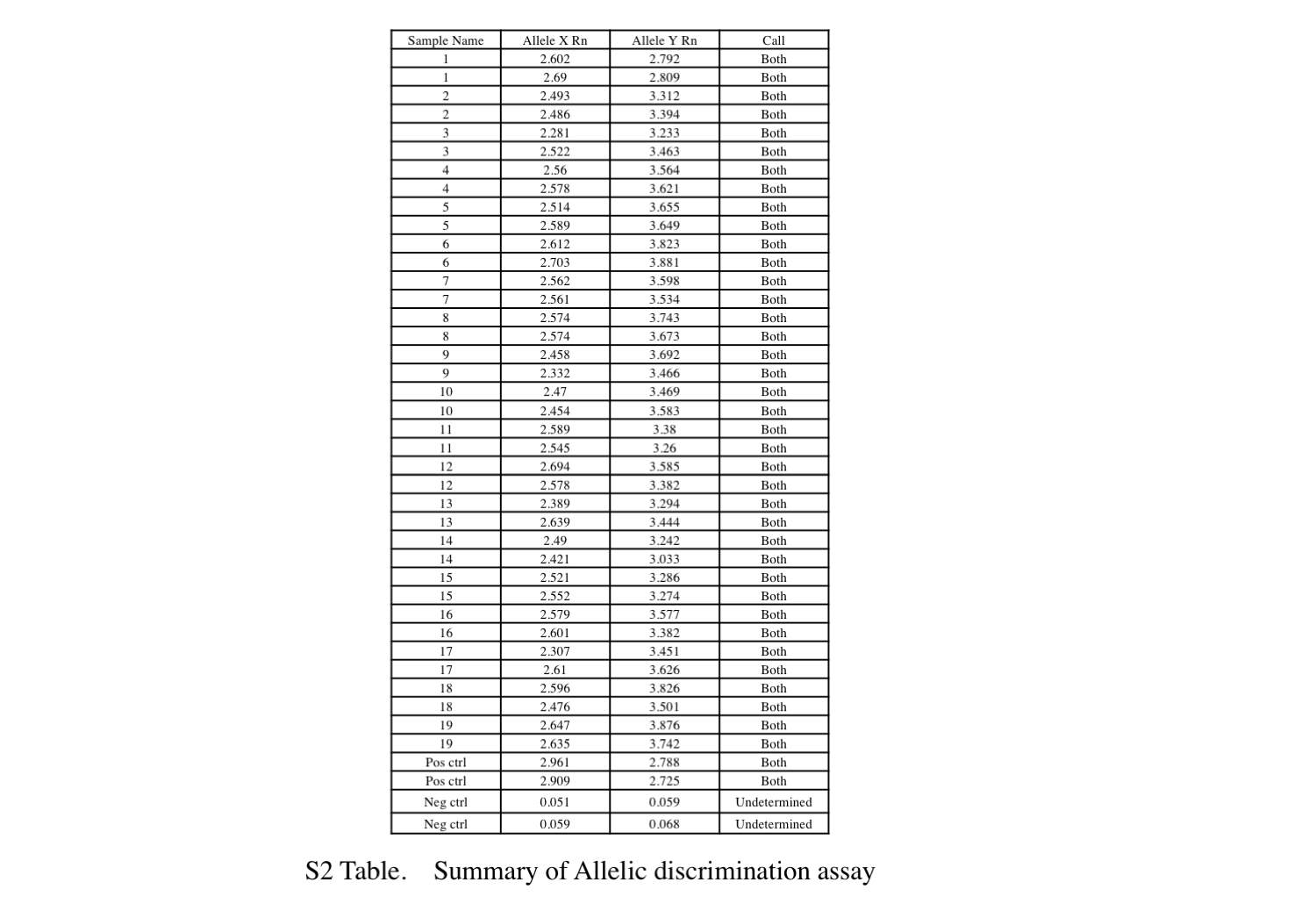

Supplement: S2 Table — (TIFF) [file pone.0178989.s002.tiff]

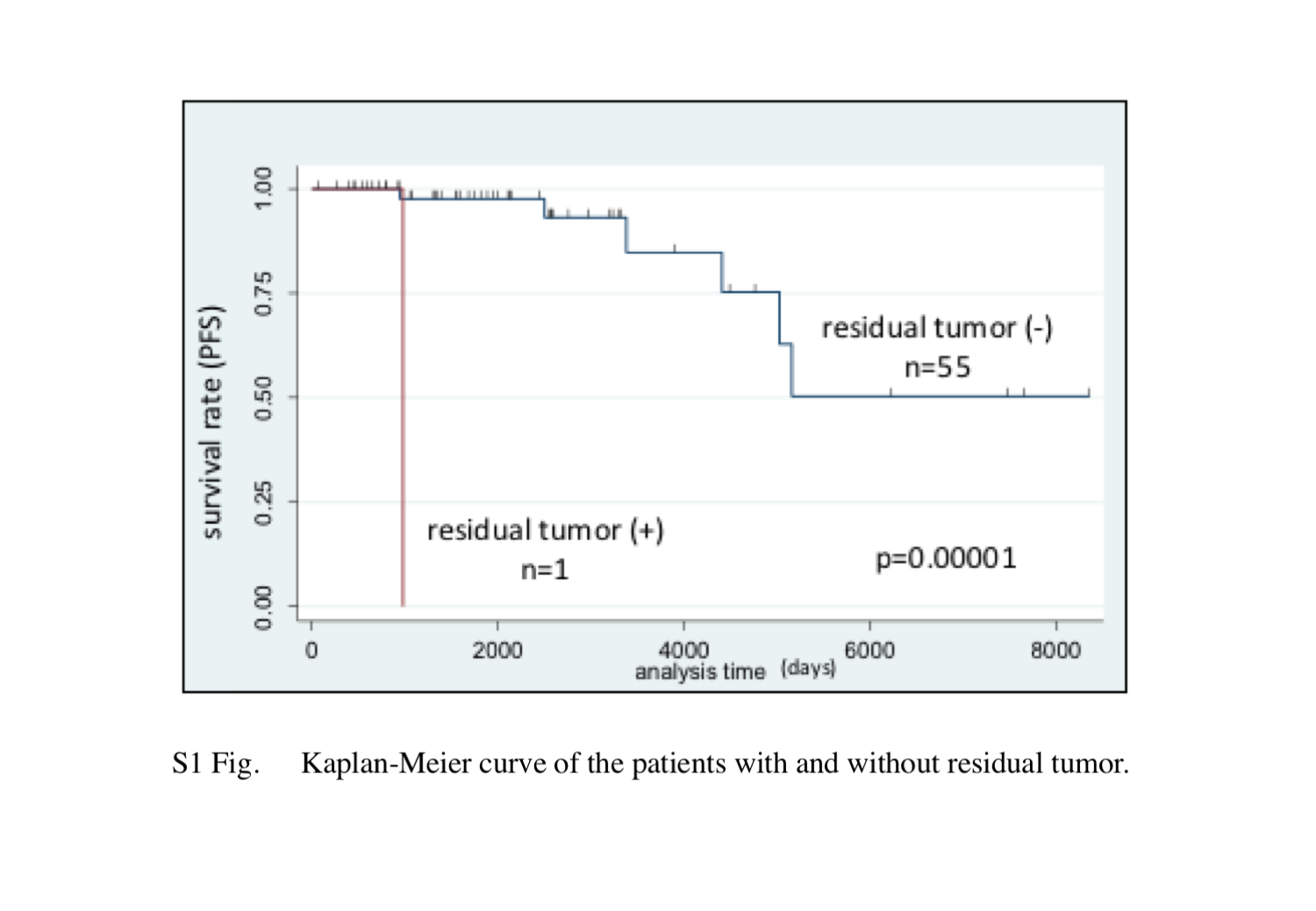

Supplement: S1 Fig — (TIFF) [file pone.0178989.s003.tiff]

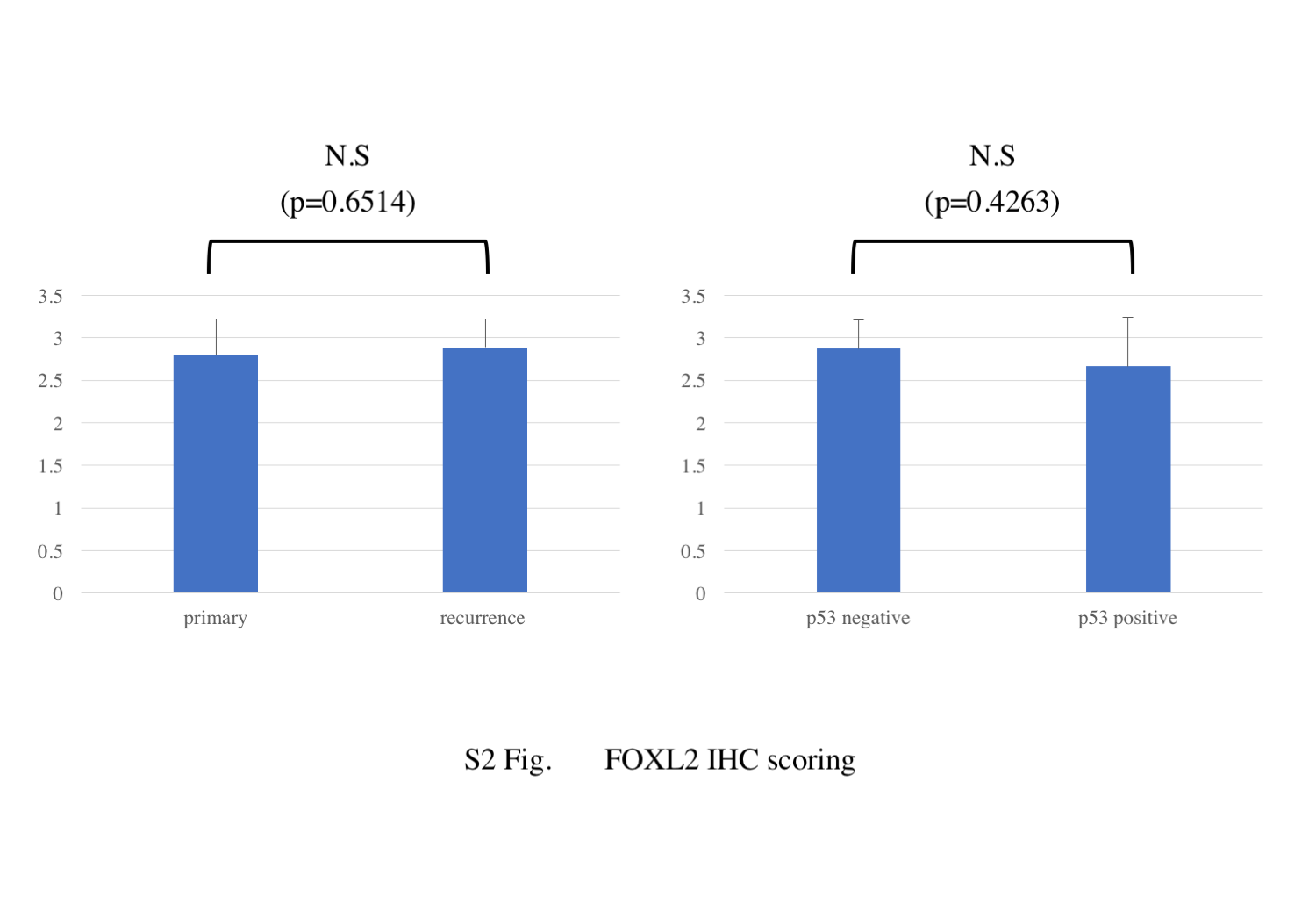

Supplement: S2 Fig — (TIFF) [file pone.0178989.s004.tiff]
